# Serum Soluble ST2 and Diastolic Dysfunction in Hypertensive Patients

**DOI:** 10.1155/2017/2714095

**Published:** 2017-05-08

**Authors:** Anca Daniela Farcaş, Florin Petru Anton, Cerasela Mihaela Goidescu, Iulia Laura Gavrilă, Luminiţa Animarie Vida-Simiti, Mirela Anca Stoia

**Affiliations:** ^1^“Iuliu Hatieganu” University of Medicine and Pharmacy, Cluj-Napoca, Romania; ^2^Emergency Clinical County Hospital, Cluj-Napoca, Romania

## Abstract

**Background:**

Echocardiographic evaluation of left ventricular (LV) structural and functional alterations in hypertension has some limitations, potentially overcome by using biomarkers. ST2, a prognostic biomarker for heart failure and myocardial infarction patients, was less studied in hypertension.

**Aim:**

To analyze the relationship between serum ST2 levels and diastolic dysfunction (DD) in hypertension.

**Method:**

We enrolled 88 hypertensive outpatients (average age 65 years, 69.3% females) in a prospective study, stratified for presence of LV hypertrophy (LVH). For each patient clinical examination, lab workup (routine and serum ST2 levels) and echocardiography were performed.

**Results:**

Hypertensive patients with LVH had higher age, pulse pressure, mean arterial pressure, and serum ST2, while having lower serum albumin than those without LVH. Serum ST2 levels correlate with parameters of LV remodeling and DD. We found that 5.3% of ST2 level variability was caused by a 1-unit variation of cardiovascular risk. We identified cut-off values for discriminating hypertension with LVH versus that without LVH and grade 2 DD versus normal diastolic performance.

**Conclusion:**

ST2 could be used as diagnostic biomarker for cardiac remodeling and altered diastolic performance in hypertension, providing additional data to echocardiography. It could represent a milestone in early detection of cardiac performance alteration.

## 1. Introduction

Hypertension is a major cardiovascular (CV) risk factor associated with a high morbidity and mortality rate as a result of the structural alterations and functional imbalance [1, 2]. Left ventricular (LV) hypertrophy (LVH) caused by pressure overload is a prognostic marker associated with a 3-fold increase in the risk for acute myocardial infarction (AMI) or death [3] and leads to heart failure (HF) [1]. Structural alterations specific to LVH may result in diastolic failure and are associated with impaired relaxation and increased LV filling pressure [4]. In contrast, concentric remodeling—that is, associated with impaired LV relaxation—does not appear to predict high filling pressure [5].

Echocardiography is a noninvasive method for identification and assessment of cardiac remodeling and its functional outcome—the diastolic dysfunction (DD) [6]. Several parameters assessed by echocardiography have a prognostic value for HF and CV death [6, 7]. Although feasible, with rates ranging from 93% for the Valsalva manoeuver to over 99% for mitral flow parameters and tissue Doppler, ambulatory echocardiographic evaluation of LV diastolic dysfunction still fails to accurately quantify it in 6–19% of cases [7, 8]. Documenting the changes in diastolic performance, especially the transition from grade 1 to grade 2, in all patients with CV disease and hypertension is difficult [6]. This is where algorithms for echocardiographic diagnosis of LVDD come into play, including state-of-the-art technology and parameters with proven diagnostic and prognostic ability.

Therefore, it is of paramount importance to identify biomarkers able to detect early stages of disease and/or monitor the progression, especially in the settings where cardiac imaging is hindered by specific comorbidities (such as obesity and lung diseases), lack of accessibility, high cost, or inexperienced operators. Such biomarkers could also be used in conjunction with other biomarkers [9, 10] or available imaging methods, thus providing additional information.

Studies have shown that ST2 is a useful biomarker to predict the evolution in both patients with [11] and without manifest CV disease but with CV risk factors (CVRF) [12, 13]. ST2 is a protein with two isoformes—a membrane-bound receptor form (ST2L) and a soluble form (sST2) secreted by cardiac myocytes exposed to stretch [14]. Interleukin-33 (IL-33) has recently been identified as the functional ligand of ST2L. Their interaction is required for the activation of the cellular cascade of events that protect the myocardium from hypertrophy and fibrosis [14, 15] by opposing the effects of angiotensin-II on myocytes. High levels of sST2 have a detrimental effect due to its action as a decoy-receptor by IL-33 neutralization, thus limiting its availability to bind to ST2L. Although in the general population, it has been shown that sST2 levels are associated with systolic blood pressure and HBP treatment [13], only few studies evaluating the correlations between ST2 levels, cardiac structural alterations, and functional changes in hypertensive patients are available.

The objectives of our study were to evaluate the correlation between ST2 levels and LVH and to assess the role of ST2 for the diagnosis of LVDD in hypertensive patients.

## 2. Patients and Methods

### 2.1. Study Group

This is an observational prospective study in which 88 hypertensive patients were enrolled from the outpatient clinic of the Cardiology Department of the Emergency Clinical County Hospital in Cluj-Napoca. Hypertension was diagnosed according to the ESH/ESC guidelines [16]—systolic blood pressure (SBP) ≥ 140 mmHg and/or diastolic (DBP) ≥ 90 mmHg. Exclusion criteria included the following: other heart diseases (ischemic HF, congenital HF, valvular HF, myocarditis, acute or chronic HF, and acute coronary syndromes), chronic inflammatory and acute infectious diseases, and pulmonary diseases (COPD, asthma, and sleep apnea). Based on LVH as assessed by echocardiography, patients were assigned to group A (*n* = 39, LVH absent) and group B (*n* = 49, LVH present). Here, we present the baseline data pertaining to the objectives of the analysis included in this manuscript. All patients signed an informed consent at enrollment before any study procedures. The study was approved by the local Ethics Committee.

### 2.2. Study Protocol

Demographic data, risk factors, and previous medical history were collected from patients' files, interview, and clinical examination, including blood pressure, weight, and height measurements. We used SBP and DBP measurements to calculate pulse pressure (PP, difference between the SBP and DBP values) and mean arterial pressure (MAP, arithmetical mean of the SBP and DBP values). Body mass index (BMI) was calculated as weight (kg)/[height (m)]^2^. Patients with a BMI below 24.99 kg/m^2^ were considered having normal weight; those with a BMI between 25 kg/m^2^ and 29.99 kg/m^2^, having overweight; and those with a BMI over 30 kg/m^2^, being with obesity. Additional CV risk was evaluated according to the ESC guidelines on hypertension [16].

Blood samples were collected, and cardiac echocardiography was performed in all enrolled patients at baseline. Blood samples for the evaluation of total, LDL, and HDL cholesterol; triglycerides; glucose; creatinine; serum albumin; uric acid; natrium; potassium; sST2 and NTproBNP were collected in the morning, after overnight fasting and after a period of 5–10-minute rest. Biochemical measurements were performed in the day of collection using routine enzymatic methods (Konelab 30, Thermo Fisher Scientific Inc., Finland). Samples for sST2 and NTproBNP were centrifuged for 15 minutes at 1000 ×g; serum was stored at −20°C until measurement. sST2 was assessed using a Soluble ST2/ILl-1R4 (human) ELISA kit (Aviscera Bioscience Inc., Santa Clara, CA 95051, USA) and an ELISA Tecan Sunrise reader. For the ST2 assay, the analytical limit of detection (sensitivity) was 5 pg/mL; intra-assay coefficients of variation (%) were 4–6% and interassay coefficients of variation (%) were 8–10%.

The plasmatic levels of NTproBNP were determined using the ELISA method (Biomedica GmbH Au, detection limit 5 pmol/L, CV intra-assay 4%, CV interassay 3.8%).

The echocardiography was performed using a 2–5 MHz probe on a Siemens Acuson X300 ultrasound machine by an experienced echocardiographer who was unaware of the medical history of the patients, in compliance with the recommendations of the American Society of Echocardiography and the European Association of Cardiovascular Imaging [17, 18], with a focus on cardiac remodeling and systolic and diastolic function. Systolic and diastolic measurements of the cardiac walls and chambers were obtained in M mode. LV mass was computed using the modified Devereux formula [6]; values > 95 g/m^2^ in women and >115 g/m^2^ in men were considered suggestive for the LVH diagnosis. LV ejection fraction (LVEF) was estimated using Simpson's biplane method. Diastolic performance was evaluated using the mitral diastolic flow pattern parameters (*E* and *A* waves [early and late diastolic peak filling velocities]), *E*/*A* waves' ratio, mitral *E* wave deceleration time (EDT), isovolumetric relaxation time (IVRT) [6], and tissue Doppler at the mitral annulus (septal and lateral). e'm (peak early diastolic mitral annulus velocity by tissue Doppler), *E*/e'm, left atrium area and volume (indexed to the body surface area) values were computed in a 4-chamber apical view. The myocardial performance index was computed as (IVCT + IVRT)/LVET (ICVT = isovolumetric contraction time, IVRT = isovolumetric relaxation time, and LVET = ejection time). Diastolic dysfunction was defined according to the American Society of Echocardiography and the European Association of Cardiovascular Imaging recommendations [18, 19] as follows: grade I (abnormal relaxation pattern)—*E*/*A* < 0.8, deceleration time (DT) > 220 ms, IVRT ≥ 100 ms, septal e' < 8 cm/s, and *E*/e'm ≤ 8; grade II (pseudonormal filling)—DT between 160 and 220 ms, *E*/*A* between 0.8 and 1.5, septal e' <8 cm/s, and *E*/e'm between 9 and 12; and grade III (restrictive filling)—*E*/A ≥ 2, DT < 160 ms, IVRT ≤ 60 ms, *E*/e'm ≥ 13 (or *E*/septal e' ≥15 and *E*/lateral e' >12).

## 3. Statistical Analysis

Statistical analysis was performed with SPSS 16. Group comparison was done using the chi-square test for categorical variables, Student test for continuous variables with normal distribution, and Mann-Whitney *U* test for continuous variables with abnormal distribution and ordinal variables. Correlation coefficients were calculated by linear regression analysis, and multiple regression analysis was applied for the analysis of the dependency between variables. Because sST2 did not have a normal distribution, we calculated its log-transformed values which were used as a dependent variable in the regression analyses. The performance of various cutoffs of sST2 for predicting the presence of LVH in patients with arterial hypertension and grade 2 diastolic dysfunction was evaluated using area under the receiver operating curve (AUC) and calculating the sensitivity and specificity. We also calculated AUC to compare the performance of sST2 in predicting the presence of grade 2 diastolic dysfunction with the one of proBNP, currently accepted as a gold standard for the assessment of ventricular dysfunction. A value of *p* < 0.05 was considered statistically significant.

## 4. Results

The study group included 88 hypertensive patients with a mean age of 65 years (range 19 years to 85 years), of which 69.3% were female. The baseline demographic characteristics are presented in Table 1. 47.72% of patients had stage 2 hypertension and 34% had stage 3 hypertension. BP was controlled in 57% of patients. Several CV risk factors (modifiable or not) were present in the study group. 90.7% of the patients had dyslipidemia: 53.5% hypercholesterolemia, 5.8% hypertriglyceridemia, and 31.4% mixed dyslipidemia. 22.1% were smokers and 51.4% had increased body mass index (27% were overweight and 24.4% obese). Diabetes was found in 34.9% of patients.

Table 2 summarizes the clinical and laboratory data of the two study groups. Patients with LVH (group B) were older than those in group A. Although differences between SBP and DBP were not statistically significant, the pulse pressure (PP) and mean arterial pressure (MAP) were significantly higher in patients from group B (with LVH) as compared to those from group A.

There were no significant differences between the two groups regarding lipid profile (total cholesterol, HDL cholesterol, LDL cholesterol, and triglycerides), serum glucose, and renal function (creatinine, urea). Albumin levels were significantly lower in patients in group B than in group A. Patients in group B had significantly higher LA area, ascending aorta diameter, interventricular septum, and LV posterior wall thickness than those in group A. Additionally, patients in group B had lower IVRT and higher *E* wave DT than patients without LVH. LV ejection fraction was normal (>50%) in all patients in the study groups.

The sST2 levels were significantly higher in patients from group B (with LVH) than in those from group A (without HVS) (36.03 ng/mL versus 29.91 ng/mL, *p* = 0.003). sST2 was negatively correlated with the degree of LVH (*r* = −0.45, *p* = 0.034).

**Figure 1:**
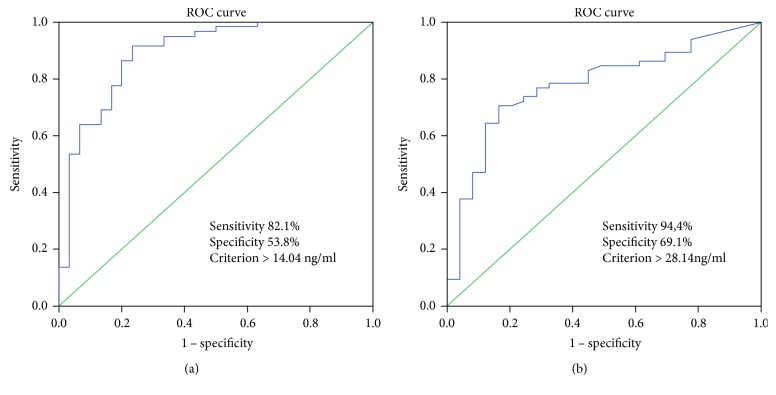
(a) ROC analysis showing the sST2 sensitivity and specificity for predicting the presence of LVH in patients with arterial hypertension. (b) ROC analysis showing ST2 sensitivity and specificity for the presence of grade 2 diastolic dysfunction in hypertensive patients.

ROC curves for sST2 showed that a cutoff value of 14.04 ng/mL had an 82.1% sensitivity and 53.8% specificity (AUC = 0.771) to discriminate between patients with and without LVH (Figure 1(a)). The sensitivity and specificity of SST2 levels of >24.18 ng/mL for the discrimination between grade 2 diastolic dysfunction and normal diastolic function in hypertensive patients were 94.4% and 69.1%, respectively (AUC = 0.721, *p* = 0.004, Figure 1(b)).

**Figure 2:**
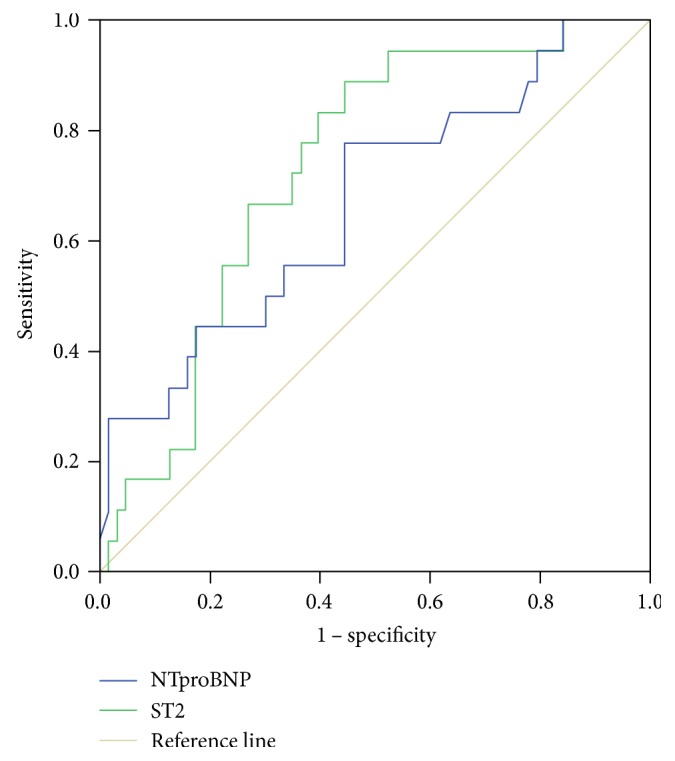
ROC analysis for the comparison between sST2 and proBNP as predictors of the presence of LVH in patients with arterial hypertension.

To further assess the performance of sST2 as a predictor of the presence of LVH in patients with arterial hypertension, we compared it with the performance of proBNP, which is considered a reference (Figure 2()). The AUC values for sST2 and proBNP were 0.732 (95% CI: 0.613–0.850) and 0.669 (95% CI: 0.524–0.814), respectively.

Correlation analysis with log-transformed sST2 as a dependent variable showed that this parameter was significantly and positively correlated with ascending aorta diameter, LV mass, end-systolic LV size, end-diastolic LV size, end-diastolic LV posterior wall, and end-diastolic interventricular septum. Regarding the diastolic function parameters, we found that sST2 was significantly and positively correlated with the *E*/*A* ratio and *E*/e'm and negatively correlated with ejection time (Table 3). No correlation was observed between sST2 and ejection fraction and left atrium area. By linear regression analysis, we found that *E*/*A*, *E*/e'm, and LVET predicted 35.6% of the variability of the sST2 levels [(*F*(3.82) = 3.940, *p* = 0.011)]. We also noticed that ST2 levels increased with the additional CV risk, 5.3% of ST2 level variability being explained by a 1-unit variation in this risk (ß = 8.3, *p* = 0.020, 95% CI 1.36–15.25) (data not shown).

By regression analysis, we showed that 33% of the variance in LV mass can be explained by a 10 mmHg variance of PP (ß = 0.2, *p* = 0.049, 95% CI 0.008–0.34).

sST2 levels increased proportionally with the presence and worsening of the diastolic dysfunction (Table 4). The highest ST2 levels were observed in stage 2 diastolic dysfunction compared to no diastolic dysfunction. In patients with diastolic dysfunction, the differences in serum ST2 levels between stage 2 versus none and stage 1 versus none, although present, did not reach statistical significance.

## 5. Discussion

Our study shows that in hypertensive patients, serum ST2 levels increase in parallel with LV mass and severity of myocardial dysfunction. Serum ST2 levels increased linearly with the degree of LVH (assessed with end-diastolic IV septum, end-diastolic LW posterior wall, and LV mass). ST2 has proven to be a useful biomarker for diagnosing LVH, with an 82.1% sensitivity and 53.8% specificity for values > 14.04 ng/mL. Ojji et al. found similar results in a cohort of hypertensive patients [20]. In 210 hypertensive patients, the authors showed that plasma ST2 levels were significantly higher in those with LVH compared to those without LVH (134.7 ng/mL versus 23.0 ng/mL) [20]. The relationship between serum ST2 levels and hypertrophic myocardium was previously shown by Sanada et al. [14] who showed that the IL-33/ST2L system is biomechanically activated and regulates cardiomyocyte hypertrophy and cardiac fibrosis.

In our study, we found serum ST2 levels had a significant and positive correlation with echocardiographic parameters of impaired relaxation (*E*/*A* ratio) and LV filling pressure (*E*/e'm ratio; correlation coefficients of 0.22 and 0.215, respectively; *p* < 0.05 for both) and had a good performance in predicting the presence of LV diastolic dysfunction in hypertensive patients. These results are in line with the ones previously reported by Ojji et al. in hypertensive patients which showed that sST2 levels were correlated with echocardiographic parameters of diastolic function—transmitral *E*/*A* ratio (Pearson correlation coefficient of 0.43) [20]. Furthermore, we showed that serum ST2 levels have the ability to discriminate between grade 2 diastolic dysfunction and normal diastolic performance, with a sensitivity of 94.4% and a specificity of 69.1% at levels higher than 24.18 ng/mL. To the best of our knowledge, these are the first published data regarding the ability of ST2 to quantify diastolic dysfunction severity in hypertensive patients and to provide a cutoff value for ST2. The recognition of LV diastolic dysfunction, regardless of systolic performance, as a distinct entity and as a negative prognostic marker has prompted a considerable amount of research for its early identification. The current state-of-the-art diagnosis involves echocardiography and is limited by technical factors (image quality, Doppler flow patterns, and availability of tissue Doppler imaging) and patient characteristics such as overweight and chest hyperinflation [18, 19]. Thus, the evaluation of ST2 may add important information to support the diagnosis of LV diastolic dysfunction. It has been previously observed that serum ST2 levels increase in parallel with the myocardial mass, and thus, it was initially hypothesized that high levels of ST2 are produced by the myocardium in response to volume and pressure overload [21]. In a study on ST2 and hemodynamic parameters in pressure overload hypertrophy (patients with aortic stenosis), Bartunek et al. [22] showed that diastolic load indeed modulates the production of ST2. Surprisingly, increased serum ST2 levels were produced not by the hypertrophied myocardium but rather by the vascular endothelial cells in response to the diastolic load [22].

We have also studied the correlation of ST2 levels with LV mass and its ability to predict the presence of LVH in our sample of hypertensive patients. ST2 was correlated with LV mass in our sample of hypertensive patients, displaying a correlation coefficient of 0.44 (*p* < 0.0001). We showed that ST2 levels of 14.04 ng/mL or above identify patients with LVH with a sensitivity of 82.1% and a specificity of 53.8%. As compared to that of proBNP, the performance of these ST2 levels were slightly improved, as shown by the AUC values higher for ST2 than for proBNP (0.732 versus 0.669). Our results confirm the previous observations reporting a higher performance of ST2 than the one of proBNP in predicting the presence of LVH in hypertensive patients [20].

Although in hypertension, LVH is an adaptive response to increased LV wall stress, the progression of myocardial hypertrophy is regulated by several hemodynamic and humoral factors. In our study, a 1 mmHg increase in pulse pressure—as a consequence of arterial stiffness induced by hypertension, age, smoking, and dyslipidemia [23]—was responsible for 3.3% of the LV mass variability. Pulse pressure was previously proven to be an independent prognostic factor for CV events in patients with hypertension [23], cost-effective, and easily quantifiable in the examination room.

We found that serum albumin levels were significantly lower in hypertensive patients with LVH and had a negative correlation with echocardiographic parameters for LV remodeling (LVH, left atrium size, and end-diastolic IV septum). The study by Ahbap et al. [24] found that serum albumin was lower in nondipper hypertensive patients compared to dippers, even for similar levels of proteinuria. Therefore, they postulated that serum albumin rather than urinary albumin excretion is an independent predictor for nocturnal dips in SBP and DBP (nocturnal SBP and DBP variability). 24 hour BP variability, daylight BP variability, and BP duration and severity are significant hemodynamic factors involved in LVH pathophysiology [25, 26].

Given all things were considered, cutoff serum ST2 values to identify LVH or quantify the level of LV diastolic dysfunction could be the milestones for the progression towards HF in hypertensive patients and identify patients with high risk of unfavorable outcome. Of course, these cutoff values would need to be validated on large population trials.

### 5.1. Limitations

Our study has several limitations. First, it enrolled a rather small group of patients; that is why we believe the relationship between ST2 and diastolic dysfunction should be evaluated in larger groups of patients with hypertension and also other CV diseases that decrease mainly the diastolic performance. The results we obtained could provide additional working hypotheses for future work. Secondly, we cannot exclude the presence of a subclinical myocardial ischemia that was unidentifiable by collecting medical history and ECG, although it is known that only myocardial remodeling secondary to acute ischemia is associated with increased serum ST2 levels [27, 28]. Thirdly, one-third of patients had diabetes—a condition characterized by a significant inflammatory status and associated with high serum ST2 levels (even in the absence of LVH) [29]—and this might have influenced our results. Additionally, we did not assess intra- and interday variability of ST2 levels measured by the assay used. To overcome the potential intraday variability, we collected all samples in the morning in fasting conditions. We could not identify any reports in the literature on the biological variability of the ST2 levels measured by the assay we used. However, assessments using a Presage ST2 assay showed that ST2 has an intraindividual coefficient of variation over a moderate time interval of 11%, lower as compared to the one of proBNP and is not influenced by the fasting or nonfasting status [30, 31].

Although with these limitations, our study offers valuable data on a real-life cohort of hypertensive patients.

## Figures and Tables

**Table 1:** Clinical and demographic data for the study group.

		Patients' number (%)	*p*
Gender	Men	27 (30.7%)	
	Women	61 (69.3%)	0.000

Location	Urban	51 (59.3%)	
	Rural	35 (40.7%)	.054

Hypertension	Stage 1	16 (18.28%)	.002^a^
	Stage 2	42 (47.72%)	.03^b^
	Stage 3	30 (34%)	.000^c^

Additional CV risk	Very high	33 (38.4%)	.04^d^
	High	38 (44.2%)	.000^e^
	Moderate	15 (17.4%)	.002^f^

Controlled hypertension	No	30 (34.5%)	
	Yes	57 (65.5%)	.000^g^

Dyslipidemia	Without	8 (9.3%)	
	Hypercholesterolemia	46 (53.5%)	
	Mixed	27 (31.4%)	
	Hypertriglyceridemia	5 (5.8%)	

Smoking	No	67 (77.9%)	
	Yes	19 (22.1%)	.001^g^

Weight status	Normal	34 (39.5%)	.04^h^
	Overweight	31 (27%)	.45^i^
	Obese	21 (24.4%)	.03^j^

Diabetes	Absent	56 (65.1%)	
	Present	30 (34.9%)	.001^k^

^a^Stage 1 versus stage 2; ^b^stage 2 versus stage 3; ^c^stage 1 versus stage 3; ^d^very high versus high; ^e^high versus moderate; ^f^very high versus moderate; ^g^no versus yes; ^h^normal versus overweight; ^i^overweight versus obese; ^j^normal versus obese; ^k^present versus absent. CV: cardiovascular.

**Table 2:** Clinical, laboratory, and echocardiographic data of patients in the two groups.

	Group A (HBP without LVH)	Group B (HBP with LVH)	*p*
Number	38	49	
Age	59.67 ± 14.47	65.17 ± 8.501	.024
Men	10 (26.3%)	16 (32.6%)	.006
Women	28 (73.7%)	33 (67.4%)	.014
SBP (mmHg)	151.00 ± 26.91	146.63 ± 24.61	.796
DBP (mmHg)	88.96 ± 11.70	95.55 ± 77.61	.413
PP	60.92 ± 11.73	62.04 ± 18.37	.002
MAP	106.16 ± 13.11	119.97 ± 18.61	.001
BMI (kg/m^2^)	30.49 ± 5.19	30.58 ± 5.43	.03
Uric acid (mg/dL)	6.57 ± 1.76	5.19 ± 2.06	.079
Creatinine (mg/dL)	0.88 ± 0.49	0.85 ± 0.28	.246
Urea (mg/dL)	31.02 ± 21.91	31.07 ± 20.31	.790
Serum albumin (g/dL)	3.5 ± 0.2	3.9 ± 1.2	.000
Serum glucose (mg/dL)	107.83 ± 23.67	107.97 ± 35.40	.338
Total cholesterol (mg/dL)	187.52 ± 58.84	201.79 ± 57.34	.862
HDL cholesterol (mg/dL)	47.05 ± 8.94	48.59 ± 13.46	.577
LDL cholesterol (mg/dL)	115.34 ± 38.83	117.38 ± 42.91	.522
Triglycerides (mg/dL)	128.73 ± 89.81	135.67 ± 68.92	.070
Na (mEq/L)	138.58 ± 2.81	139.32 ± 8.24	.476
K (mEq/L)	4.28 ± 0.48	4.38 ± 0.53	.850
ST2 (ng/mL)	29.91 ± 24.12	36.03 ± 29.91	.003
Ascending aorta (mm)	26.46 ± 8.97	31.46 ± 5.38	.002
Left atrium size (mm)	31.13 ± 8.98	34.81 ± 7.55	.056
Left atrium area (mm)	5.79 ± 9.55	9.74 ± 10.63	.003
Left atrium area index (mm)	21.32 ± 8.56	28.47 ± 7.21	.001
End-systolic interventricular septum (mm)	9.46 ± 5.37	13.92 ± 5.78	.002
End-diastolic interventricular septum (mm)	9.71 ± 1.53	13.01 ± 1.56	.000
End-systolic LV posterior wall (mm)	10.78 ± 4.77	13.88 ± 5.13	.012
End-diastolic LV posterior wall (mm)	10.01 ± 1.43	12.11 ± 2.32	.000
End-systolic LV size (mm)	24.77 ± 14.07	28.30 ± 11.17	.224
End-diastolic LV size (mm)	42.52 ± 6.11	44.57 ± 8.95	.306
Right ventricle size (mm)	26.35 ± 4.28	26.28 ± 9.11	.074
Stroke volume (mL)	13.92 ± 22.96	37.37 ± 34.70	.001
LV mass (g/m^2^)	145.4 ± 62.2	262.9 ± 83.6	.000
LV mass index	44.95 ± 85.30	164.15 ± 128.93	.000
IVRT (ms)	127.96 ± 72.47	98.38 ± 49.03	.009
EDT (ms)	211.72 ± 120.06	218.64 ± 71.65	.005

HBP: hypertension; LVH: left ventricular hypertrophy; SBP: systolic blood pressure; DBP: diastolic blood pressure; PP: pulse pressure; MAP: mean arterial pressure; BMI: body mass index; LV: left ventricle; IVRT: isovolumetric relaxation time; EDT: mitral *E* wave deceleration time.

**Table 3:** Correlation of clinical/echocardiographic parameters with log-transformed ST2 and diastolic dysfunction.

Log-transformed ST2	Correlation coefficient	*p*
BMI	0.21	.056
SBP	0.127	.237
DBP	0.03	.981
PP	0.178	.097
MAP	0.071	.509
CVAR	0.253	.023
Ascending aorta	0.212	.048
Left atrium area	0.16	.21
End-diastolic interventricular septum	0.51	.000
End-diastolic LV posterior wall	0.40	.03
End-diastolic LV size	0.28	.02
End-systolic LV size	0.35	.01
LV mass index	0.44	.000
Ejection fraction	0.119	.268
*E*/*A*	0.22	.03
*E*/e'm	0.215	.02
Ejection time	−0.259	.015

Diastolic dysfunction	Correlation coefficient	*p*

Ascending aorta	0.212	.048
Left atrium area	0.16	.21
End-diastolic interventricular septum	0.51	.000
End-diastolic LV posterior wall	0.40	.03
End-diastolic LV size	0.28	.02
End-systolic LV size	0.35	.01
LV mass index	0.44	.000
Peak *E*/*A* ratio	0.22	.03
Myocardial performance index	0.245	.012
ST2	0.273	.006
*E*/e'm	0.547	.000
Ejection time	0.235	.015

HBP: hypertension; LVH: left ventricular hypertrophy; SBP: systolic blood pressure; DBP: diastolic blood pressure; PP: pulse pressure; MAP: mean arterial pressure; BMI: body mass index; LV: left ventricle; CVAR: cardiovascular additional risk; *A*: late diastolic mitral peak filling velocity; *E*: early diastolic mitral peak filling velocity; e'm: peak early diastolic mitral annulus velocity by tissue Doppler.

**Table 4:** Serum sST2 levels of patients according to the presence of diastolic dysfunction and cardiovascular risk.

		ST2 (ng/mL)	*p*
E/e'm	≤8	30.67 ± 22.18	0.03^a^
	9–12	33.34 ± 27.56	0.002^b^
	≥13	42.16 ± 39.51	.000^c^

Diastolic dysfunction	Absent	27.06 ± 12.97	.048^d^
	Grade 1/mild	30.67 ± 21.06	.016^e^
	Grade 2/moderate	43.54 ± 17.49	.008^f^

Diabetes	Absent	32.76 ± 20.61	.002^g^
	Present	39.16 ± 40.98	

Additional CV risk	Moderate	23.66 ± 15.73	.020^h^
	High	31.58 ± 20.96	.04^i^
	Very high	40.16 ± 28.03	.026^j^

^a^ *E*/e'm ≤ 8 versus *E*/e'm = 9–12; ^b^*E*/e'm = 9–12 versus *E*/e'm ≥ 13; ^c^*E*/e'm ≤ 8 versus *E*/e'm ≥ 13; ^d^absent versus grade 1; ^e^grade 1 versus grade 2; ^f^absent versus grade 2; ^g^absent versus present; ^h^moderate versus high; ^i^high versus very high; ^j^moderate versus very high; CV: cardiovascular.
